# Identification of MRI-Derived Structural Biomarkers in Female Alzheimer’s Disease Subjects Using CAT12 and Mimics: Effects of Voxel Geometry on Biomarker Estimation

**DOI:** 10.3390/life16091490

**Published:** 2026-09-06

**Authors:** Devang Nilesh Thakur, Tarun Goswami

**Affiliations:** 1 Department of Neuroscience, Cell Biology & Physiology, Wright State University, Dayton, OH 45435, USA; thakur.48@wright.edu; 2 Department of Biomedical, Industrial, and Human Factors Engineering, Wright State University, Dayton, OH 45435, USA

**Keywords:** Alzheimer’s disease, Magnetic Resonance Imaging (MRI), structural biomarkers, cortical thickness, voxel-based morphometry, CAT12, Materialise Mimics

## Abstract

Alzheimer’s disease (AD) is the leading cause of dementia and disproportionately affects women, who experience a higher lifetime risk and more rapid structural brain changes than men. Reliable imaging biomarkers are essential for detecting these changes, although their estimation may be influenced by voxel geometry, image resolution, and segmentation methodology. In this study, magnetic resonance imaging (MRI) scans from 40 female participants with AD obtained from the Alzheimer’s Disease Neuroimaging Initiative (ADNI) database were analyzed using the Computational Anatomy Toolbox 12 (CAT12), implemented within Statistical Parametric Mapping 12 (SPM12), and Materialise Mimics to identify structural biomarkers associated with neurodegeneration. Cortical thickness, gray matter (GM), white matter (WM), cerebrospinal fluid (CSF), and total intracranial volume (TIV) were quantified, while Brain Parenchymal Volume (BPV) and Brain Parenchymal Fraction (BPF) were calculated to assess global brain tissue preservation. The analyses demonstrated characteristic AD-related changes, including cortical thinning, GM loss, ventricular enlargement, and increased CSF volume. Significant correlations among cortical thickness, tissue volumes, BPV, and BPF further supported their complementary role in characterizing disease-related structural changes. Overall, these findings suggest that MRI-derived measures of cortical thickness, tissue volumes, BPV, and BPF provide useful structural biomarkers of AD and emphasize the importance of considering voxel geometry and segmentation methodology when evaluating neuroimaging biomarkers.

## 1. Introduction

Alzheimer’s disease (AD) is the most common cause of dementia and is considered one of the biggest public health challenges of the twenty-first century. The disease is characterized by progressive cognitive decline, memory loss, executive dysfunction, and eventually a loss of independence. As life expectancy continues to increase around the world, the prevalence of AD is also rising, creating a significant burden on patients, caregivers, and healthcare systems. Current estimates indicate that more than 55 million people worldwide are living with dementia [1], with AD accounting for roughly 60–70% of all cases. Since pathological changes can begin many years before noticeable clinical symptoms appear, there is growing interest in identifying reliable biomarkers that can detect neurodegeneration during the earliest stages of disease development [2,3,4].

**Figure 1:**
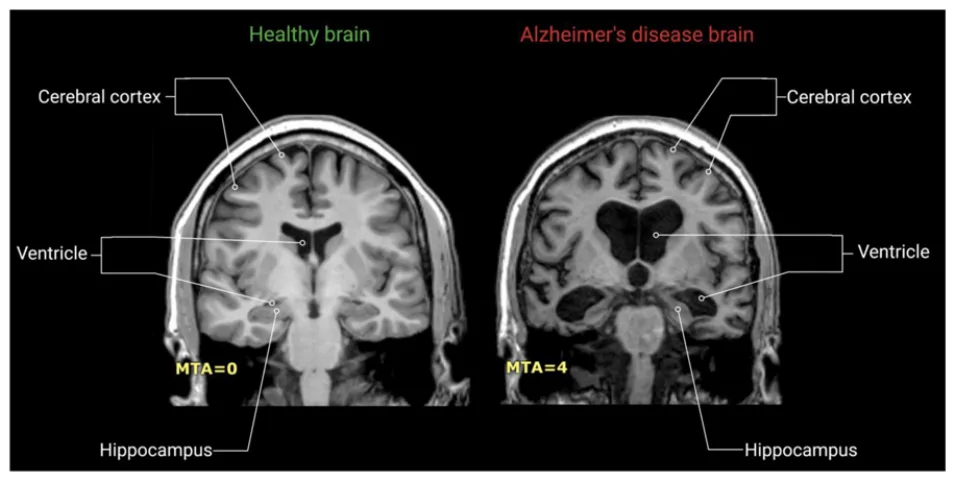
Structural brain changes associated with Alzheimer’s disease (AD). Comparison of a healthy brain (**left**) and an AD brain (**right**). AD is characterized by hippocampal atrophy, cortical thinning, and ventricular enlargement. Progressive neurodegeneration results in reduced hippocampal volume, shrinkage of the cerebral cortex, and expansion of the ventricular system. Medial temporal lobe atrophy (MTA) is commonly used to assess hippocampal degeneration, where MTA = 0 indicates no visible atrophy and MTA = 4 indicates severe hippocampal volume loss. Adapted from van Oostveen and de Lange (2021) [4].

Magnetic resonance imaging (MRI) has become one of the most important non-invasive techniques for studying AD. Structural MRI provides detailed visualization of brain anatomy and allows researchers to quantitatively assess neurodegenerative changes associated with disease progression. Many studies have shown that AD is linked to characteristic alterations in brain structure, including cortical thinning, GM loss, hippocampal atrophy, ventricular enlargement, and expansion of CSF spaces [3,5,6]. These structural changes are considered valuable imaging biomarkers because they reflect the neuronal loss and tissue degeneration that occur throughout the disease process. As a result, MRI-derived biomarkers are increasingly being used for early diagnosis, disease staging, monitoring progression, and evaluating treatment outcomes [4,7,8]. Figure 1 illustrates representative structural brain changes associated with AD, including cortical thinning, hippocampal atrophy, and ventricular enlargement.

Among the various MRI biomarkers that have been investigated, cortical thickness has shown considerable promise for detecting early neurodegenerative changes. Although hippocampal atrophy is a well-established biomarker of AD, hippocampal volume was not directly quantified in the present study and is discussed primarily for context. Previous studies have reported significant thinning in temporoparietal, frontal, and medial temporal regions in individuals with AD compared to cognitively healthy controls [3,9]. Dickerson et al. found that cortical thinning within a characteristic “AD cortical signature” was strongly associated with disease severity and could even be detected in asymptomatic individuals with amyloid pathology [3]. Likewise, hippocampal atrophy has long been recognized as one of the earliest and most reliable structural indicators of AD because of the hippocampus’s essential role in learning and memory formation [10,11]. Enlargement of the lateral ventricles has also been identified as a robust marker of disease progression, reflecting the loss of surrounding brain tissue over time [5,12].

In addition to regional biomarkers, global volumetric measurements provide useful information about overall brain integrity. GM, WM, CSF, and TIV are commonly used to quantify structural changes associated with neurodegeneration. AD is generally characterized by reductions in GM volume along with expansion of CSF spaces and ventricular structures [3,5]. Derived measures such as BPV, calculated as the sum of GM and WM volumes, and BPF, calculated as the ratio of BPV to TIV, offer integrated assessments of overall brain tissue preservation [7,13]. These biomarkers may provide additional sensitivity for measuring global atrophy and evaluating disease severity, especially when combined with more traditional morphometric measures.

Sex differences have become an increasingly important topic in AD research. Women account for nearly two-thirds of AD cases and have a higher lifetime risk of developing dementia compared with men [14,15,16]. Emerging evidence suggests that women may experience different patterns of neurodegeneration, including faster cortical thinning, increased hippocampal vulnerability, and altered biomarker progression trajectories [14,17,18]. Several neuroimaging studies have reported significant sex-specific differences in structural MRI biomarkers, indicating that female-focused investigations may reveal disease characteristics that are not fully captured in mixed-sex studies [14,18]. Understanding these differences is important for developing more personalized approaches to diagnosis and treatment and for improving outcomes in populations that are disproportionately affected by AD.

The accuracy and reproducibility of MRI-derived biomarkers depend heavily on image acquisition parameters and segmentation methods. Factors such as voxel geometry, slice thickness, spatial resolution, and preprocessing strategies can significantly influence measurements of cortical thickness, tissue volumes, and ventricular size [19,20]. Partial-volume effects caused by larger voxels may blur tissue boundaries, while isotropic resampling procedures can modify anatomical representations and affect morphometric estimates. Consequently, quantitative biomarker values may vary across software platforms and processing pipelines, even when the same MRI datasets are analyzed [2,20]. Understanding the impact of these methodological factors is essential for improving reproducibility and enhancing the clinical usefulness of MRI-based biomarkers.

CAT12, which is implemented within the Statistical Parametric Mapping (SPM12) framework, is one of the most widely used neuroimaging toolboxes for automated morphometric analysis [19]. CAT12 performs tissue segmentation, cortical surface reconstruction, cortical thickness estimation, voxel-based morphometry, and quality-control assessment through a largely automated workflow. Previous research has shown that CAT12 produces highly reproducible measurements and performs similarly to established neuroimaging platforms such as FreeSurfer, while also allowing efficient processing of large imaging datasets [2,6]. In addition to conventional volumetric outputs, CAT12 generates surface topology metrics such as Euler number, gyrification indices, and defect-area estimates, which may provide additional information about structural alterations associated with neurodegenerative diseases [6].

Voxel-based morphometry (VBM) is a whole-brain, voxel-wise neuroimaging method used to assess regional differences in brain tissue volume. In CAT12, VBM involves tissue segmentation and spatial normalization, allowing voxel-wise comparison of gray matter and other tissue characteristics across brain regions. This approach provides quantitative information about regional structural differences and complements surface-based measures such as cortical thickness.

In contrast, Materialise Mimics 26.0 uses a surface-guided segmentation approach that focuses on preserving native image geometry. Through thresholding, region growing, mask editing, and manual refinement, Mimics enables detailed three-dimensional reconstruction of anatomical structures while reducing the influence of automated resampling procedures. This approach can provide highly detailed visualization of ventricular systems, cortical surfaces, and other structures relevant to AD pathology. Although Mimics generally requires more operator involvement and processing time than automated voxel-based methods, its ability to preserve anatomical detail may improve the characterization of subtle structural abnormalities and serve as a useful validation tool for automated segmentation pipelines.

Despite the widespread use of MRI biomarkers in AD research, relatively few studies have directly investigated how voxel geometry affects biomarker estimation across fundamentally different segmentation approaches. In addition, limited research has focused specifically on female AD cohorts while simultaneously comparing automated voxel-based morphometry and surface-guided segmentation techniques. The effects of voxel geometry on biomarkers such as BPV, BPF, cortical thickness, and ventricular morphology are still not fully understood. Addressing this gap is important because even small differences in biomarker estimates may influence diagnostic interpretation, longitudinal monitoring, and assessments of disease progression.

Therefore, the objective of this study was to investigate MRI-derived structural biomarkers in female AD subjects obtained from the ADNI database. Using both CAT12 and Materialise Mimics, cortical thickness, GM volume, WM volume, CSF volume, and TIV were evaluated to determine how voxel geometry and segmentation methodology influence biomarker estimation. Additionally, derived biomarkers including BPV and BPF were calculated and analyzed to assess their potential usefulness in characterizing neurodegeneration. By combining automated voxel-based morphometry with surface-guided segmentation, this study aims to identify robust MRI biomarkers of AD and improve our understanding of the methodological factors that can influence quantitative neuroimaging measurements.

## 2. Materials and Methods

### 2.1. Study Population and MRI Data Acquisition

Magnetic resonance imaging (MRI) data were obtained from the Alzheimer’s Disease Neuroimaging Initiative (ADNI) database. Forty female subjects diagnosed with AD were selected for analysis based on the availability of high-quality T1-weighted structural MRI scans. All images were acquired using Magnetization Prepared Rapid Gradient Echo (MPRAGE) sequences with approximately 1.0 mm isotropic voxel resolution. The ADNI database was chosen because it provides standardized neuroimaging datasets collected using harmonized imaging protocols across multiple research sites, allowing for reliable comparison of structural biomarkers among subjects [21].

### 2.2. MRI Preprocessing

Prior to segmentation, all MRI scans underwent preprocessing to ensure image quality and consistency. Images were visually inspected for motion artifacts, acquisition errors, and gross anatomical abnormalities. Structural MRI datasets were converted into formats compatible with the processing software and prepared for subsequent morphometric analysis. Particular attention was given to maintaining voxel geometry and image integrity because previous studies have demonstrated that voxel dimensions can significantly influence volumetric and cortical thickness measurements [19,22].

### 2.3. CAT12 Processing and Biomarker Extraction

**Figure 2:**
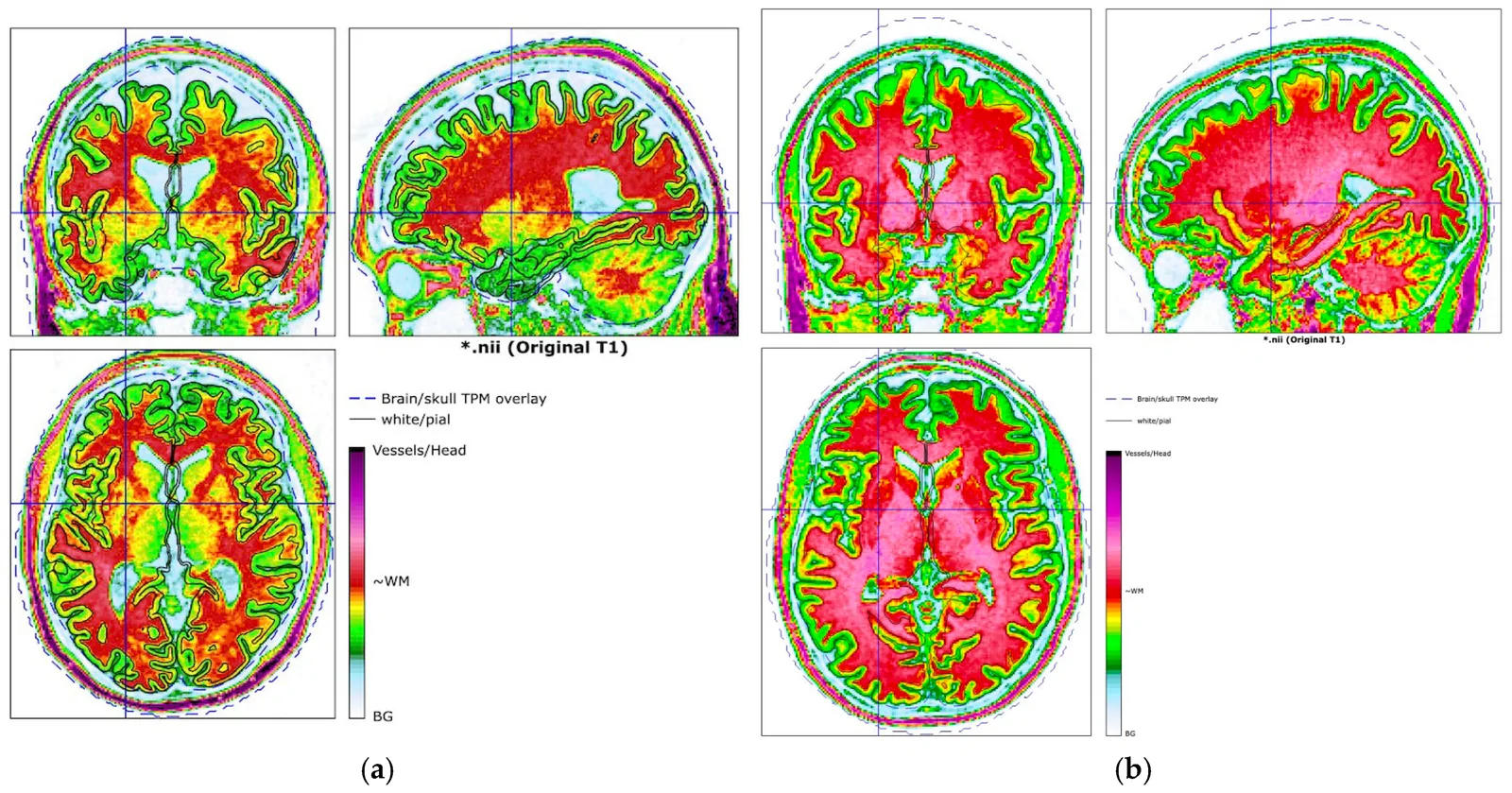
CAT12 voxel-based segmentation outputs for an AD subject (**a**) and CN subject (**b**), showing GM/WM/CSF classification and surface boundaries. The asterisk (*) indicates the original T1-weighted image (.nii).

All MRI datasets were processed using Computational Anatomy Toolbox (CAT12.9, r2577) implemented within Statistical Parametric Mapping (SPM25, version 25.01.02) in MATLAB R2025b. CAT12 performed automated tissue segmentation, bias-field correction, skull stripping, spatial normalization, and cortical surface reconstruction [2,6]. The software automatically classified brain tissue into GM, WM, and CSF compartments. Representative CAT12 voxel-based segmentation outputs are shown in Figure 2. Following segmentation, volumetric and morphometric biomarkers were extracted, including:

• GM Volume (GM)
• WM Volume (WM)
• CSF Volume (CSF)
• TIV
• Mean Cortical Thickness.

In addition, CAT12 generated quality-control metrics and surface reconstruction outputs that were reviewed to verify segmentation accuracy.

### 2.4. Mimics Segmentation and Three-Dimensional Reconstruction

To complement the automated CAT12 workflow, MRI datasets were also analyzed using Materialise Mimics 26.0. Mimics utilizes a surface-guided segmentation approach that preserves native image geometry and enables detailed three-dimensional visualization of anatomical structures. Segmentation was performed using thresholding, region-growing algorithms, mask editing, and manual refinement procedures. Three-dimensional reconstructions of brain tissues were generated to visualize cortical morphology, ventricular enlargement, and structural changes associated with AD. Mimics outputs were used to validate anatomical features identified through CAT12 and to assess the influence of segmentation methodology on biomarker estimation.

### 2.5. Biomarker Calculations

In addition to directly measured biomarkers, several derived biomarkers were calculated to provide integrated measures of brain tissue preservation and atrophy.

BPV was calculated as:

BPV = GM + WM

where GM represents gray matter volume and WM represents white matter volume.

BPF was calculated as:

BPF = BPV/TIV

where TIV represents total intracranial volume.

Tissue percentages were calculated using the following equations:

CSF% = (CSF/TIV) × 100

GM% = (GM/TIV) × 100

WM% = (WM/TIV) × 100

These derived measurements were used to quantify the relative distribution of intracranial tissues and evaluate structural brain preservation among AD subjects.

### 2.6. Statistical Analysis

Descriptive statistics were calculated for all biomarkers, including CSF volume, GM volume, WM volume, TIV, cortical thickness, BPV, and BPF. Mean values, standard deviations, and ranges were examined to characterize variability within the study population. Pearson correlation analysis was performed to investigate relationships among MRI-derived biomarkers. Correlation coefficients (r) were calculated between all variables, including tissue volumes, tissue percentages, cortical thickness, BPV, and BPF. The resulting correlation matrix was visualized using a correlation heatmap. Positive correlations were represented by warmer colors, while negative correlations were represented by cooler colors. The heatmap was used to identify relationships among biomarkers and determine which structural measures exhibited the strongest associations with indicators of brain atrophy and tissue preservation in AD.

### 2.7. Workflow Overview

**Figure 3:**
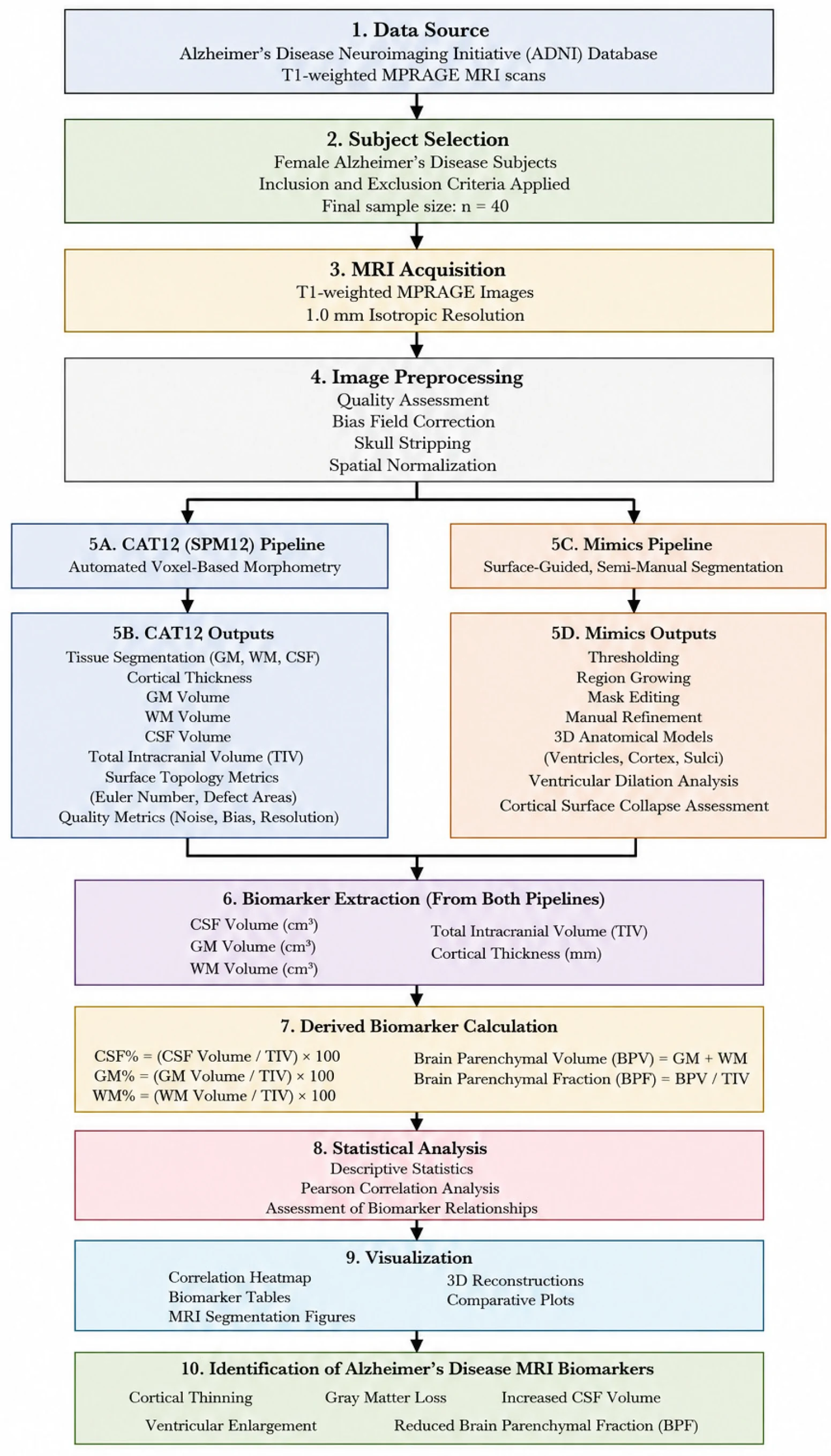
Study workflow illustrating MRI acquisition from the ADNI database, preprocessing, CAT12 and Mimics segmentation, biomarker extraction, BPV/BPF calculation, statistical analysis, and identification of AD biomarkers.

The overall study workflow consisted of MRI acquisition from the ADNI database, preprocessing of structural MRI scans, automated biomarker extraction using CAT12, anatomical validation and three-dimensional reconstruction using Mimics, calculation of derived biomarkers (BPV and BPF), and statistical analysis through Pearson correlation and heatmap visualization. This combined approach enabled assessment of both voxel-based and surface-guided segmentation techniques for identifying structural biomarkers associated with AD. An overview of the study workflow is shown in Figure 3.

## 3. Results

**Figure 4:**
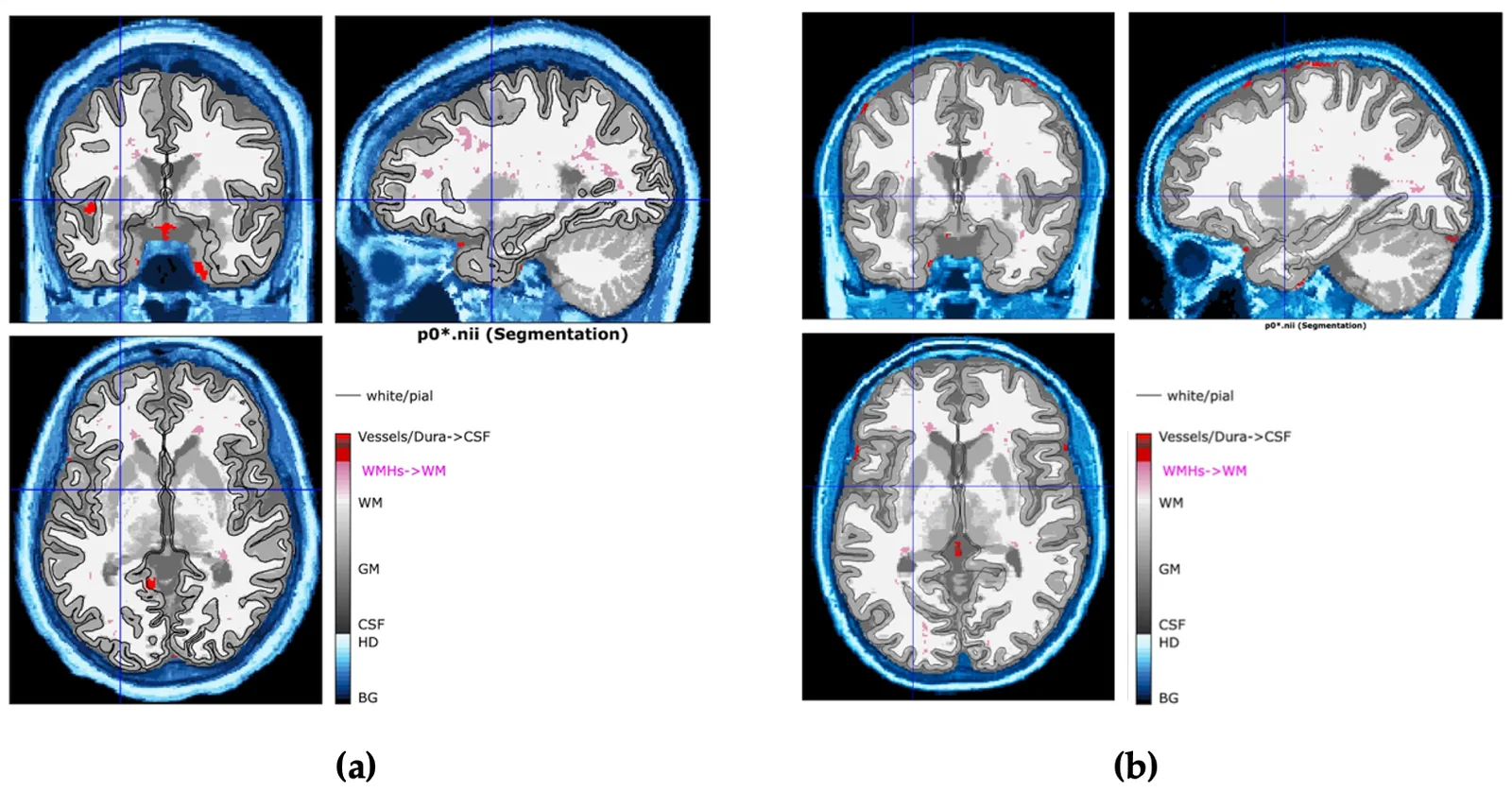
CAT12 segmentation maps for AD subject 941_S_6962 (**a**) and CN subject 014_S_6076 (**b**). The AD subject shows enlarged ventricles, reduced cortical thickness, and expanded CSF spaces compared to the CN subject. The asterisk (*) indicates the original T1-weighted image filename associated with the segmentation output.

Across the ADNI AD subjects analyzed, both CAT12 and Mimics revealed convergent but method-specific patterns of neurodegeneration that directly support the identification of structural MRI biomarkers for AD. CAT12 consistently detected reduced GM volume, lower cortical thickness, and enlarged CSF spaces in AD subjects compared to cognitively normal (CN) reference scans, with GM volumes frequently falling below 500 cm^3^ and cortical thickness values clustering around 2.1–2.3 mm. Mean BPF values ranged from approximately 0.61 to 0.78, reflecting varying degrees of brain tissue preservation across subjects. These voxel-based outputs also showed elevated noise levels, higher Euler numbers, and larger surface defect areas in AD subjects, reflecting the increased anatomical irregularity caused by atrophy. These findings are illustrated in Figure 4.

**Figure 5:**
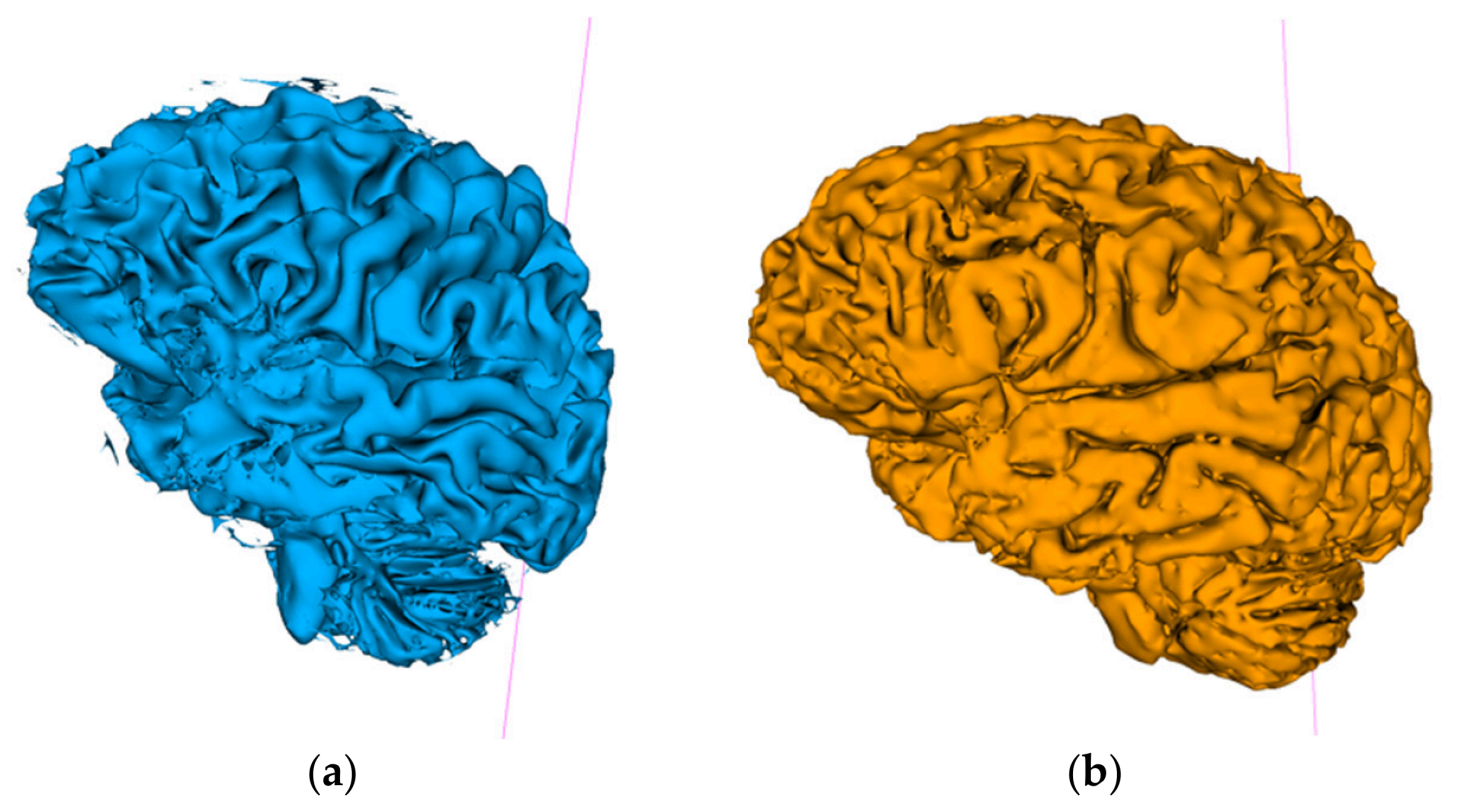
Mimics 3D reconstructions for the same subjects. (**a**) AD model (blue), showing sulcal widening and cortical surface collapse; (**b**) CN model (orange), showing preserved cortical morphology.

Mimics segmentation further reinforced these findings by revealing pronounced ventricular dilation, sulcal widening, and collapse of cortical surfaces, particularly in the medial temporal lobe and perisylvian regions, areas known to degenerate early in AD (Figure 5). Importantly, Mimics required substantially more manual correction for AD subjects due to sharper CSF–GM boundaries and fragmented cortical and ventricular contours, highlighting how disease-driven geometry interacts with slice-dependent segmentation.

When comparing volumetric outputs across pipelines, both methods consistently identified enlarged ventricles, reduced cortical volumes, cortical thinning, and increased CSF spaces in AD subjects, but Mimics tended to exaggerate ventricular size and capture finer surface irregularities that CAT12′s resampling partially smooths. Together, these results demonstrate that voxel geometry and segmentation strategy meaningfully influence biomarker estimates, and that integrating voxel-based (CAT12) and slice-based (Mimics) morphometry provides a more complete characterization of AD-related structural decline. This dual-pipeline approach strengthens biomarker detection by revealing which morphometric features remain stable across methods (e.g., ventricular enlargement) and which are sensitive to voxel resolution (e.g., cortical thickness and fine anatomical boundaries), thereby improving the reliability of MRI-based AD diagnostics. The MRI biomarker measurements for the AD subjects are summarized in Table 1.

**Table 1:** MRI Biomarker Measurements of AD Subjects.

Subject ID	CSF (cm^3^)	GM (cm^3^)	WM (cm^3^)	TIV (cm^3^)	CSF %	GM %	WM %	Thickness (mm)	BPV (cm^3^)	BPF
168_S_6843	267	495	396	1158	23.1	42.7	34.2	2.24 ± 0.50	891	0.769
135_S_6840	372	589	407	1368	27.2	43.1	29.7	2.54 ± 0.64	996	0.728
129_S_6763	332	522	372	1226	27.1	42.6	30.3	2.21 ± 0.61	894	0.729
061_S_1789	360	580	410	1355	26.5	42.8	30.7	2.33 ± 0.57	990	0.731
123_S_6825	431	510	393	1334	32.3	38.3	29.5	2.26 ± 0.57	903	0.677
037_S_6230	448	536	382	1366	32.8	39.2	28	2.28 ± 0.57	918	0.672
098_S_6658	415	558	381	1355	30.7	41.2	28.1	2.28 ± 0.55	939	0.693
033_S_10049	495	568	468	1522	32.5	36.7	30.8	2.20 ± 0.52	1036	0.681
056_S_10042	410	530	380	1320	31	40	29	2.18 ± 0.52	910	0.689
036_S_6231	589	553	352	1494	25.3	37	37.6	2.18 ± 0.54	905	0.606
016_S_7039	290	829	407	1226	23.7	43.1	33.2	2.35 ± 0.65	988	0.698
033_S_10249	363	484	415	1262	28.8	38.4	32.9	2.02 ± 0.50	899	0.712
061_S_6738	300	510	390	1200	25	42.5	32.5	2.27 ± 0.58	900	0.75
067_S_7033	385	522	447	1353	28.4	38.6	33	2.14 ± 0.50	969	0.716
049_S_16783	350	560	400	1310	27	41.5	31.5	2.25 ± 0.55	960	0.733
033_S_10147	298	586	461	1345	22.1	43.6	34.3	2.24 ± 0.55	1047	0.778
011_S_10044	410	569	445	1424	28.8	40	31.2	2.31 ± 0.55	1014	0.712
023_S_3493	320	600	420	1340	24	45	31	2.30 ± 0.60	1020	0.761
022_S_6013	341	489	358	1188	28.7	41.1	30.1	2.35 ± 0.56	847	0.713
002_S_1018	343	557	401	1317	26	42.3	30.4	2.26 ± 0.56	958	0.727
003_S_1059	455	543	416	1334	34.1	40.7	31.2	2.26 ± 0.57	959	0.719
005_S_0814	301	563	396	1311	23	42.9	30.2	2.25 ± 0.55	959	0.731
006_S_0653	491	539	421	1339	36.7	40.3	31.4	2.26 ± 0.58	960	0.717
007_S_1304	258	568	390	1305	19.8	43.5	29.9	2.25 ± 0.54	958	0.734
010_S_10871	421	548	412	1329	31.7	41.2	31	2.26 ± 0.57	960	0.722
011_S_10026	346	557	402	1318	26.3	42.3	30.5	2.26 ± 0.56	959	0.728
011_S_10257	375	553	406	1322	28.4	41.8	30.7	2.26 ± 0.56	959	0.725
014_S_10351	283	565	394	1308	21.6	43.2	30.1	2.25 ± 0.55	959	0.733
018_S_0335	477	541	419	1337	35.7	40.5	31.3	2.26 ± 0.58	960	0.718
018_S_5074	340	558	401	1317	25.8	42.4	30.4	2.26 ± 0.56	959	0.728
023_S_0139	311	561	397	1312	23.7	42.8	30.3	2.26 ± 0.55	958	0.73
023_S_1262	364	555	404	1320	27.6	42	30.6	2.26 ± 0.56	959	0.727
023_S_1289	337	558	401	1316	25.6	42.4	30.5	2.26 ± 0.56	959	0.729
027_S_0404	484	540	420	1338	36.2	40.4	31.4	2.26 ± 0.58	960	0.718
027_S_10800	309	562	397	1312	23.6	42.8	30.3	2.25 ± 0.55	959	0.731
027_S_1082	360	555	404	1320	27.3	42	30.6	2.26 ± 0.56	959	0.727
027_S_1385	445	545	415	1332	33.4	40.9	31.2	2.26 ± 0.57	960	0.721
031_S_1209	351	556	403	1318	26.6	42.2	30.6	2.26 ± 0.56	959	0.728
032_S_1101	469	542	418	1336	35.1	40.6	31.3	2.26 ± 0.58	960	0.719

**Figure 6:**
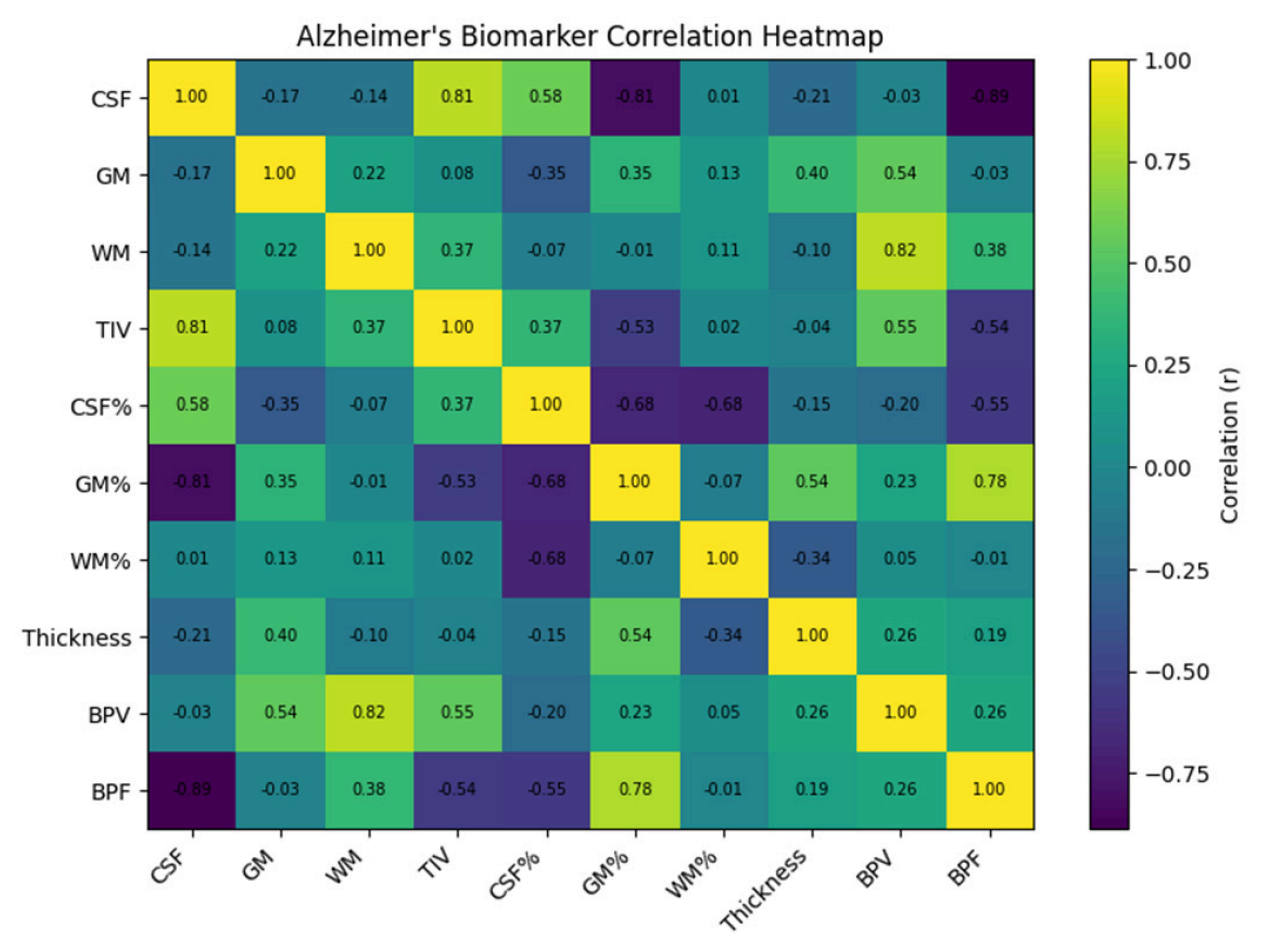
Correlation heatmap illustrating relationships among MRI-derived biomarkers, including CSF, GM, WM, TIV, cortical thickness, BPV, and BPF. Warmer colors represent positive correlations, whereas cooler colors represent negative correlations. Strong negative correlations were observed between CSF-related measures and BPF, while positive correlations were identified between GM measures, cortical thickness, and brain parenchymal preservation.

To further investigate the relationships among MRI-derived biomarkers, Pearson correlation analysis was performed using volumetric, morphometric, and derived measurements obtained from the AD cohort. The resulting correlation matrix was visualized as a heatmap (Figure 6), allowing identification of associations between CSF, GM, WM, TIV, cortical thickness, BPV, and BPF.

The correlation heatmap revealed several notable relationships among structural biomarkers. CSF volume demonstrated a strong positive correlation with TIV (r = 0.81) and a strong negative correlation with BPF (r = −0.89), indicating that increasing CSF volume was associated with reduced preservation of brain tissue. Similarly, CSF percentage showed negative correlations with both GM percentage and BPF, suggesting that expansion of CSF spaces accompanies progressive tissue loss and cerebral atrophy.

GM measurements exhibited positive associations with markers of structural preservation. GM percentage showed a strong positive correlation with BPF (r = 0.78), while cortical thickness demonstrated moderate positive correlations with GM percentage and BPF. These findings indicate that subjects with greater cortical preservation generally maintained higher proportions of brain parenchymal tissue.

WM volume also contributed substantially to overall brain preservation. A strong positive correlation was observed between WM volume and BPV (r = 0.82), reflecting the importance of WM integrity in maintaining total brain tissue volume. Positive relationships were additionally observed between BPV and BPF, supporting the use of these derived biomarkers as indicators of global structural preservation.

Overall, the heatmap identified a consistent pattern in which biomarkers associated with tissue loss and ventricular expansion correlated negatively with measures of brain preservation, whereas cortical thickness, GM volume, and parenchymal measures demonstrated positive relationships. These findings support the utility of BPF, BPV, cortical thickness, and tissue composition metrics as complementary structural biomarkers for characterizing AD-related neurodegeneration.

## 4. Discussion

The purpose of this study was to evaluate MRI-derived structural biomarkers of AD using CAT12 and Materialise Mimics while examining the influence of voxel geometry and segmentation methodology on biomarker estimation. Across the forty female AD subjects analyzed, both software platforms consistently identified characteristic features of neurodegeneration, including reduced GM volume, cortical thinning, ventricular enlargement, and increased CSF volume. Although the two segmentation approaches differed in their processing methods, both produced findings consistent with established structural changes reported in the AD literature.

One of the most consistent findings observed in this study was reduced cortical thickness among AD subjects. Mean cortical thickness values generally ranged between 2.1 and 2.3 mm, reflecting widespread cortical atrophy. These findings are consistent with the work of Dickerson et al. [3], who identified a characteristic pattern of cortical thinning in temporoparietal and medial temporal regions that strongly correlated with disease severity. Similarly, Keuss et al. [9] reported that cortical thinning in AD signature regions may serve as a sensitive marker of neurodegeneration and disease progression. The cortical thickness values observed in the present study further support the role of cortical atrophy as a major imaging biomarker of AD.

In addition to cortical thinning, significant alterations in brain tissue composition were observed. Subjects frequently demonstrated reduced GM volumes accompanied by increased CSF volumes and enlarged ventricular spaces. These findings are consistent with previous studies showing that neuronal loss in AD is associated with progressive GM reduction and expansion of CSF-filled spaces [3,5,6]. Nestor et al. [5] reported that ventricular enlargement is one of the most reliable structural indicators of disease progression and may reflect cumulative tissue loss throughout the brain. Similar ventricular expansion was observed in both CAT12 and Mimics segmentations within the present study.

The use of Materialise Mimics provided additional insight into the anatomical consequences of neurodegeneration. Three-dimensional reconstructions clearly demonstrated ventricular dilation, widening of cortical sulci, and collapse of cortical surfaces in many subjects. These visual findings correspond closely with previous neuroimaging studies that describe ventricular enlargement and cortical surface deformation as hallmark features of AD [4,12]. While CAT12 efficiently quantified these structural changes through automated segmentation, Mimics allowed direct visualization of the anatomical abnormalities underlying the numerical measurements.

A major addition to this study was the calculation of BPV and BPF. These derived biomarkers were included to provide a more comprehensive assessment of overall brain tissue preservation. Subjects with lower BPF values generally exhibited increased CSF percentages, lower cortical thickness measurements, and reduced GM volumes. Similar findings have been reported by Frisoni et al. [7,13], who suggested that global measures of brain atrophy may improve the assessment of disease burden when used alongside regional biomarkers such as hippocampal volume and cortical thickness. Because BPF accounts for both brain tissue volume and intracranial volume, it may provide a useful measure of overall neurodegeneration.

Correlation analysis further strengthened the interpretation of these biomarkers. The correlation heatmap demonstrated a strong negative relationship between CSF volume and BPF, indicating that subjects with greater CSF expansion generally exhibited lower levels of preserved brain tissue. Positive correlations were observed between cortical thickness, GM measurements, BPV, and BPF. These relationships are biologically expected because progressive neuronal loss results in cortical thinning, GM reduction, and enlargement of CSF spaces. The heatmap therefore provides quantitative evidence that multiple MRI-derived biomarkers are interconnected and collectively reflect the neurodegenerative processes underlying AD.

Another important finding was the influence of segmentation methodology on biomarker estimation. Although both CAT12 and Mimics identified similar patterns of structural decline, differences were observed in the magnitude of ventricular enlargement and cortical surface irregularities. Previous studies comparing segmentation platforms have reported similar variability among CAT12, FreeSurfer, and ANT-based analyses [2,20,23]. The findings of the present study suggest that voxel geometry and preprocessing choices can influence quantitative biomarker values and should be carefully considered when comparing results across studies or imaging centers [2,22].

Several limitations should be acknowledged. First, the study included only female AD subjects, limiting direct comparison with male populations. However, this female-focused approach was intentional because women account for nearly two-thirds of AD cases and often exhibit distinct neurodegenerative trajectories [14,15,16]. Second, the sample size was limited to forty subjects, and larger cohorts would improve statistical power. Third, cognitive assessment scores were not included in the present analysis, preventing direct evaluation of the relationship between MRI-derived structural biomarkers and cognitive performance. Therefore, although the observed structural measures are consistent with neurodegeneration, their association with the severity of cognitive impairment could not be established. Future studies should integrate standardized cognitive assessments with MRI-derived biomarkers to determine whether regional and global structural measures correlate with clinical measures of disease severity. Finally, the study focused exclusively on structural MRI and did not incorporate complementary biomarkers such as amyloid PET imaging, tau imaging, or CSF protein analysis.

Future studies should expand the cohort size and investigate how BPV and BPF change across different stages of AD. Additional comparisons involving FreeSurfer, ANTs, and other segmentation platforms may further improve understanding of methodological variability. Longitudinal analyses would also provide valuable information regarding the ability of these biomarkers to track disease progression over time.

Overall, the results demonstrate that cortical thickness, GM volume, ventricular enlargement, CSF expansion, BPV, and BPF represent promising structural biomarkers of AD. The combination of CAT12 and Mimics provided complementary information regarding both quantitative measurements and anatomical visualization, supporting a more comprehensive characterization of AD-related neurodegeneration.

## 5. Conclusions

This study evaluated MRI-derived structural biomarkers of AD using both CAT12 and Materialise Mimics in a cohort of forty female AD subjects obtained from the ADNI database. Across both segmentation platforms, characteristic features of neurodegeneration were consistently identified, including cortical thinning, GM loss, increased CSF volume, and ventricular enlargement. These findings are consistent with established structural changes associated with AD and support the use of MRI as a valuable tool for biomarker identification and disease characterization.

The comparison between CAT12 and Mimics demonstrated that voxel geometry and segmentation methodology can influence biomarker estimation, particularly for cortical thickness measurements and the visualization of fine anatomical structures. While CAT12 provided efficient automated extraction of morphometric measurements, Mimics offered detailed three-dimensional visualization of anatomical changes associated with neurodegeneration. Together, these approaches provided complementary information regarding both quantitative biomarker assessment and anatomical interpretation.

In addition to traditional MRI biomarkers, this study incorporated BPV and BPF as measures of global brain tissue preservation. Correlation analysis revealed meaningful relationships among CSF volume, GM volume, cortical thickness, BPV, and BPF, demonstrating that these biomarkers collectively reflect the structural consequences of AD. In particular, increased CSF volume was associated with reduced brain parenchymal preservation, while greater cortical thickness and GM volume were associated with higher BPF values.

Overall, the results suggest that cortical thickness, GM volume, ventricular enlargement, BPV, and BPF represent promising structural biomarkers of AD. Furthermore, the findings emphasize the importance of considering voxel geometry and segmentation strategy when interpreting MRI-derived measurements. Future studies involving larger cohorts, longitudinal imaging, and additional neuroimaging modalities may further improve the reliability and clinical utility of these biomarkers for early detection, disease monitoring, and therapeutic evaluation in AD.

## Data Availability

The data analyzed in this study are available from the Alzheimer’s Disease Neuroimaging Initiative (ADNI) database (https://adni.loni.usc.edu, accessed on 14 December 2025), upon registration and approval in accordance with ADNI data access policies. Individual participant-level data cannot be redistributed by the authors in accordance with the ADNI Data Use Agreement.

## Abbreviations

AD	Alzheimer’s disease
ADNI	Alzheimer’s Disease Neuroimaging Initiative
BPF	Brain Parenchymal Fraction
BPV	Brain Parenchymal Volume
CAT12	Computational Anatomy Toolbox 12
CSF	Cerebrospinal Fluid
GM	Gray Matter
MRI	Magnetic Resonance Imaging
SPM12	Statistical Parametric Mapping 12
TIV	Total Intracranial Volume
VBM	Voxel-Based Morphometry
WM	White Matter
